# Gemcitabine and Cisplatin as Neo-Adjuvant for Cholangiocarcinoma Patients Prior to Liver Transplantation: Case-Series

**DOI:** 10.3390/curroncol29050290

**Published:** 2022-05-17

**Authors:** Maen Abdelrahim, Hadeel Al-Rawi, Abdullah Esmail, Jiaqiong Xu, Godsfavour Umoru, Fahad Ibnshamsah, Ala Abudayyeh, David Victor, Ashish Saharia, Robert McMillan, Ebtesam Al Najjar, Doaa Bugazia, Maryam Al-Rawi, Rafik M. Ghobrial

**Affiliations:** 1 Section of GI Oncology, Department of Medical Oncology, Houston Methodist Cancer Center, Houston, TX 77030, USA; hadeel.h.alrawi@gmail.com (H.A.-R.); aesmail@houstonmethodist.org (A.E.); 2 Cockrell Center of Advanced Therapeutics Phase I Program, Houston Methodist Research Institute, Houston, TX 77030, USA; 3 Weill Cornell Medical College, New York, NY 14853, USA; asaharia@houstonmethodist.org (A.S.); rrmcmillan@houstonmethodist.org (R.M.); rmghobrial@houstonmethodist.org (R.M.G.); 4 Faculty of Medicine, University of Jordan, Amman 11942, Jordan; mariamalrawi12@gmail.com; 5 Cancer Clinical Trials, Houston Methodist Research Institute, Houston, TX 77030, USA; 6 JC Walter Jr Center for Transplantation and Sherrie and Alan Conover Center for Liver Disease and Transplantation, Houston, TX 77030, USA; dwvictor@houstonmethodist.org; 7 Center for Outcomes Research, Houston Methodist Research Institute, Houston, TX 77030, USA; sxu@houstonmethodist.org; 8 Department of Pharmacy, Houston Methodist Cancer Center, Houston, TX 77030, USA; goumoru@houstonmethodist.org; 9 Medical Oncology, King Fahd Specialist Hospital, Buraydah 52366, Saudi Arabia; fahad.ibnshamsah@kfsh.med.sa; 10 Faculty of Medicine, Imam Abdulrahman Bin Faisal University, Dammam 34212, Saudi Arabia; 11 Section of Nephrology, Division of Internal Medicine, The University of Texas MD Anderson Cancer Center, Houston, TX 77030, USA; aabudayyeh@mdanderson.org; 12 Faculty of Medicine and Health Sciences, University of Science and Technology, Sanaa 15201, Yemen; ebtsam.hasan89@gmail.com; 13 Faculty of Medicine, University of Tripoli, Tripoli 22131, Libya; dogazia89@gmail.com

**Keywords:** hepatocellular carcinoma, gemcitabine, cisplatin, immunotherapy, liver transplantation, cholangiocarcinoma

## Abstract

Background: The management of cholangiocarcinoma is continually reviewed on a current evidence basis to develop practice guidelines and consensus statements. However, the standardized treatment guidelines are still unclear for cholangiocarcinoma patients who are listed for liver transplantation. We aimed to validate and evaluate the potential efficacy of chemotherapy combination of Gemcitabine and Cisplatin as a neo-adjuvant treatment for cholangiocarcinoma patients before liver transplantation. Methods: In this prospective case series, patients with locally advanced, unresectable, hilar, or intrahepatic cholangiocarcinoma with no evidence of extrahepatic disease or vascular involvement were treated with a combination of neoadjuvant gemcitabine and cisplatin with no radiation. All patients included received chemotherapy prior to being listed for liver transplantation at a single cancer center according to an open-labeled, and center-approved clinical management protocol. The primary endpoints were the overall survival and recurrence-free survival after liver transplantation. Results: Between 1 March 2016, and 15 March 2022, 10 patients (8 males and 2 females) with a median age of 62.71(interquartile range: 60.02–71.87) had a confirmed diagnosis of intrahepatic or hilar cholangiocarcinoma and underwent liver transplantation. Median days of neoadjuvant therapy for a given combination of gemcitabine and cisplatin were 181 (IRQ: 120–250). Nine patients (90%) were reported with no recurrence or metastasis, and only 1 patient had confirmed metastasis (10%); days for metastasis after transplantation were 612 for this patient. All patients received a combination of gemcitabine and cisplatin as neo-adjuvant while awaiting liver transplantation. The median days of follow-up were 851 (813–967). Overall survival was 100% (95% CI 100–100%) at both years one and two; 75% (95% CI 13–96%) at years three to five. One patient died at eight hundred and eighty-five days. No adverse events were reported after liver transplantation including the patient who was confirmed with recurrence. Conclusions: Our finding demonstrated that neo-adjuvant gemcitabine and cisplatin with no radiation prior to liver transplantation resulted in excellent outcomes for patients with cholangiocarcinoma.

## 1. Introduction

The second most common primary liver cancer after hepatocellular Carcinoma is cholangiocarcinoma which is a rare tumor [1]. However, its worldwide incidence is increasing, and the five-year survival rate is only 25% despite tumor resection. Tumor recurrence is the major reason for mortality in 53–79% of patients. The recurrence is usually localized in 83% of patients and occurs within two years post tumor resection [2,3,4,5,6,7]. In this highly infiltrative cancer, there is insufficient local tumor control with resection, and survival rates are suboptimal even with systemic chemotherapy, regional ablative options, and radiation. Due to the tumor site, size, or multifocality, most patients present with unresectable disease.

Liver transplantation for patients with unresectable cholangiocarcinoma has been previously investigated [8,9,10]. Outcomes for intrahepatic cholangiocarcinoma are poor in comparison with hepatocellular carcinoma, with five years overall survival rate of 25% [11,12,13]. Consequently, most centers consider intrahepatic cholangiocarcinoma a contraindication to liver transplant. Hilar cholangiocarcinoma initially had poor outcomes with liver transplants. However, per the United Network of Organ Sharing (UNOS) database, hilar cholangiocarcinoma patients who have undergone pre-transplant anticancer therapy have significant survival outcomes in contrast with transplanted patients for incident disease. The improved survival rate for cholangiocarcinoma patients treated with liver transplants preceded by neoadjuvant chemoradiation has been reported in 14 studies [13,14,15,16,17,18].

The five-year survival rate after liver transplant was reported to be up to 65% by a multicenter study. Thus, liver transplantation became a preferred strategy for patients with early-stage and unresectable hilar cholangiocarcinoma. However, only incidental or misdiagnosed intrahepatic cholangiocarcinoma tumors identified on pathology can undergo liver transplantation [19,20,21]. Patients with cholangiocarcinoma who might benefit from transplantation have also been shown to have a good response to neoadjuvant therapy.

Pre-transplant therapy has been shown to reduce disease recurrence in both intrahepatic and hilar cholangiocarcinoma [15,19,20,21]. The outcomes of liver transplantation in 48 patients with intrahepatic cholangiocarcinoma who received locoregional therapy or didn’t receive neoadjuvant chemotherapy were analyzed by a retrospective, multicenter international study [10]. Advanced (>2 cm) intrahepatic cholangiocarcinoma) five-year overall survival was found to be up to 45% while early intrahepatic cholangiocarcinoma (≤2 cm) was up to 65%. These results indicate that for small, solitary intrahepatic cholangiocarcinoma, a liver transplant might be indicated if pre-transplant therapy is not feasible. Regarding multifocal, larger tumors the outcome of neoadjuvant chemotherapy remains significantly undetermined. The option of treating cholangiocarcinoma and hepatocellular carcinoma via liver transplant has been actively evolving with promising outcomes in addition, other related options to liver transplant are keep evolving such as the main loco-regional therapies that can be used to reduce the tumor burden in locally advanced cases who are not LR candidates, including transarterial chemoembolization (TACE), selective internal radiation therapy (SIRT), radiofrequency ablation (RFA), and photodynamic therapy (PDT) should be mentioned. These treatments, in fact, can control local tumors and avoid systemic treatment side effects and allow these patients to eventually be resected or transplanted [19,22,23,24,25]. As far as we know, no previous reports have studied the effect of gemcitabine and cisplatin as neo-adjuvant therapy for cholangiocarcinoma patients prior to liver transplantation. This study reports our experience and outcomes with chemotherapy combination of gemcitabine and cisplatin as a neo-adjuvant treatment for cholangiocarcinoma patients prior to liver transplantation.

## 2. Methods

### 2.1. Participants

This study is based on the clinical management of patients with intrahepatic and hilar cholangiocarcinoma at our institution. We have been providing liver transplantation to eligible patients with intrahepatic or hilar cholangiocarcinoma since 2016. The clinical management protocol requires the usage of a combination of gemcitabine and cisplatin as neo-adjuvant for intrahepatic or hilar cholangiocarcinoma and encourages biological stability or response for choosing patients for liver transplantation.

With regards to our liver transplantation protocol, locally advanced intrahepatic or hilar cholangiocarcinoma was defined as a solitary tumor if greater than 2 cm in diameter or if the multifocal disease was confined to the liver without radiological evidence of extrahepatic, macrovascular, or lymph node involvement. Patients were selected if there was tumor regression on neoadjuvant therapy or if they had shown six months of disease stability. A multidisciplinary case review by the tumor board was carried out to choose potentially appropriate candidates composed of medical oncologists, transplant surgeons, radiation oncologists, hepatologists, pathologists, and interventional radiologists. A formal screening meeting and comprehensive evaluation of medical history were carried out on the chosen candidates by our tumor board. Acceptable patients were then brought up for formal liver transplantation evaluation and listing. In order to be listed, patients should have had a biopsy or cytology confirming cholangiocarcinoma and had unresectable tumors either due to site or underlying liver disease after six months of neoadjuvant therapy. The feasibility of tumor resections was based on a majority decision after evaluation by GI oncologists and liver transplant surgeons from Houston Methodist Hospital.

For liver transplantation to be qualified under clinical administration protocol, the malignant tumor should be found in those patients and proofed by imaging characteristics (proportionate with intrahepatic cholangiocarcinoma (CEA) and serum cancer antigen (CA)19-9 concentrations greater than 100 U/mL), or biopsy confirming malignancy and unresectable disease [based on technical analysis or underlying liver disease, supported response for at least six months with the combination of gemcitabine and cisplatin neo-adjuvant therapy] assessed by MRI or CT Scan. In addition, patients must have had no evidence of extrahepatic disease, at least six months of sustained response after the most recent therapy, reduction of transperitoneal tumor aspiration, or biopsy, and control of biliary sepsis. Patients were excluded if there was a presence of extrahepatic metastases, lymph node involvement, invasion or encasement of major hepatic vascular structures, invasion of extrahepatic structures, and perforation of the visceral peritoneum. Furthermore, patients had to undergo a complete medical and psychosocial work-up. There were no age or morbidity limitations and only adult patients (18 years or older) were considered if they met medical criteria for liver transplantation. Transplanted patients in this series were recorded between 1 March 2016, and 15 March 2022. Attending GI oncologists and surgeons were consulted to assess the risks of using orphan livers. At transplant, recipients were consented to by the transplanting surgeon and were informed of the risks of receiving a transplant, including portal vein or hepatic artery thrombosis and primary non-function, with additional agreement obtained in cases where donors were at a high risk of disease transmission. The collection of data from recipients was carried out in compliance with the Houston Methodist Institutional Review Board approved protocol (IRB ID: PRO00032826).

### 2.2. Gemcitabine plus Cisplatin as Neo-Adjuvant Treatment

**Figure 1:**
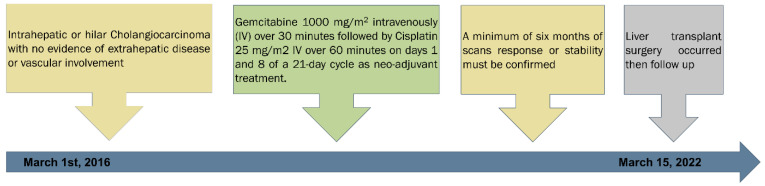
Schema of the treatment timeline of gemcitabine and cisplatin as neo-adjuvant for cholangiocarcinoma patients prior to liver transplantation.

All of the patients received gemcitabine plus cisplatin regimen as neoadjuvant therapy. The regimen consisted of gemcitabine 1000 mg/m^2^ which was administered intravenously (IV) over 30 min followed by cisplatin 25 mg/m^2^ IV over 60 min on days 1 and 8 of a 21-day cycle. All patients on the liver transplantation waiting list were monitored for any changes for at least six months. Patients who progressed within this time frame were excluded from the study Figure 1.

With regards to follow-up, patients were monitored for any new events, or any obstructive symptoms such as jaundice, pruritus, clay-colored stools, dark urine, or any other systemic symptoms including, weight loss, fever, and night sweats. We also performed physical examinations and repeated tumor markers such as CA19-9 and CEA. MRI and PET scans were also performed and evaluated for pertinent clinical findings.

### 2.3. Follow Up

Patients were followed up every month with a complete blood cell count and prothrombin time test, as well as liver function tests for alanine aminotransferase (ALT), aspartate aminotransferase (AST), bilirubin, albumin, and carcinoembryonic antigen CEA. In addition, radiographic tests such as a liver contrast-enhanced CT or MRI, as well as a chest CT and/or bone scan, were carried out every six to nine weeks to evaluate the treatment response. The tumor response was assessed using the Modified Response Evaluation Criteria in Solid Tumors (mRECIST). The overall survival was measured from the beginning of the first treatment cycle to death or the last visit, whichever came first. Patients who were lost to follow-up were tracked until the last date they were known to be alive, and those who survived were censored until the data cutoff date of December 2021 Figure 1.

### 2.4. Statistical Analysis

For CCA recurrence after transplantation, frequencies, and proportions as well as categorical variables, such as demographic and clinical data, were reported, while continuous variables were presented as the median and interquartile range or mean (standard deviation [SD]). For categorical variables, the Chi-square or Fisher’s exact tests were used, while for continuous variables, the Kruskal-Wallis test or unpaired t-test was used. The Kaplan-Meier curves were utilized to show overall patient survival and CCA-free survival. The log-rank test was used to compare differences across groups. Stata version 17.0 was used for all analyses (StataCorp LLC, College Station, TX, USA). A *p*-value of 0.05 was considered to be statistically significant [26,27].

## 3. Results

Between 1 March 2016, and 15 March 2022. 10 patients (8 males and 2 females) with a median age of 62.71 (interquartile range: 60.02–71.87) were diagnosed with cholangiocarcinoma Table 1. All of the patients included in this study had been subjected to liver transplantation for intrahepatic cholangiocarcinoma. Among these patients 10% were Asian, 10% were black, and 80% were Caucasian. Also, 20% of patients identified as Hispanic or Latino.

All patients received the combination of gemcitabine and cisplatin for 181 (120.00–250.00) days. Most patients (*n* = 9) had no recurrence or metastasis. Tumor recurrence was only reported in one patient in the study group (10%) and occurred 612 days after liver transplant.

**Figure 2:**
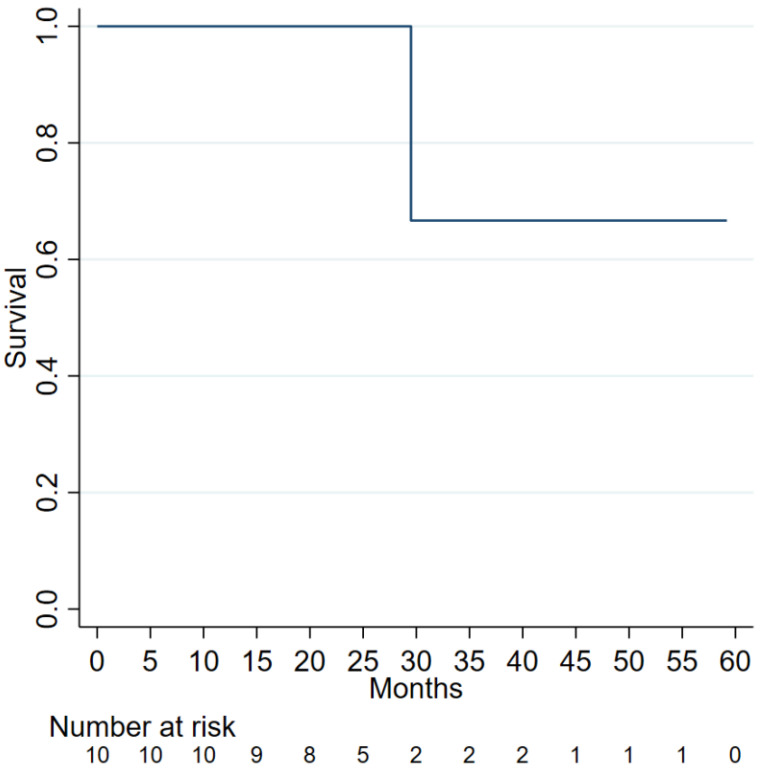
Overall survival was 100% (95% CI 100–100) at one year and two years, respectively. In the third year, the overall survival was 75% (95% CI 13–96%). One patient died at eight hundred and eighty-five days. This patient died due to acute renal failure superimposed on stage 3 chronic kidney disease.

All patients in the current study received the combination of gemcitabine and cisplatin as neoadjuvant therapy while waiting for liver transplantation. After a median follow-up of 851 (813–967), overall survival (OS) was 100% (95% CI 100–100), respectively. In the third to fifth year, the overall survival rate was 75% (95% CI 13–96%) Figure 2. At the last follow-up, 1 patient died at 885 days. No significant adverse effects were observed since it wasn’t reported after liver transplantation, including in the patient who was confirmed with recurrence.

Patients in the study group concerning the gemcitabine–cisplatin combination regimen with IHCCA or HCCA were also compared separately evaluating the risk of recurrence or rejection. No significant differences were observed in the baseline characteristics of patients in either group. Among the 10 patients in the study group, 3 patients were classified as Hilar CCA subgroup whereas the IHCCA subgroup was composed of 7 patients. The tumor recurrence rate/rejection was relatively higher in IHCCA subjects being occupying one patient who was on the regimen for almost four months. The rejection occurred two years after the transplant and he died a few months later (Table 2).

Regarding the duration of treatment for other IHCCA patients who received gemcitabine plus cisplatin as a neo-adjuvant treatment for cholangiocarcinoma prior to liver transplantation, the shortest period was on three months while the longest period was nine months. None of these patients experienced tumor rejection or recurrence. Details are shown in (Table 1). Concerning Hilar CCA, there were no reports of recurrence or rejection, and patients were on the regimen for at least six months, with one patient completing up to two years of therapy. After transplantation patients were discharged home and followed up for median days of 851.00 (813.00–967.00).

Our results indicate the practicality of this approach and highlight the demand for a large-scale, multi-center, prospective trial of neoadjuvant therapy followed by liver transplantation for intrahepatic cholangiocarcinoma in the future.

## 4. Discussion

The number of patients diagnosed with cholangiocarcinoma continues to rise. Thus, to assess the outcome of therapy with gemcitabine and cisplatin as neo-adjuvant for cholangiocarcinoma, we analyzed patients with stable intrahepatic or hilar cholangiocarcinoma before they had liver transplantation and found an overall survival rate of 100% in both years one and two compared to 75% (95% CI 13–96%) at years three to five. In the third year, tumor recurrence was reported in only one patient in 10% of the study group, and this case was reported 612 days after the liver transplant Figure 2. The selection criteria may recognize samples of intrahepatic cholangiocarcinoma patients who may benefit from a liver transplant. These results recommend that therapy response and the stability of the tumor at each time could work as a substitute marker for approving tumor biology for a liver transplant.

Not many previous studies have proposed a possible dominance of pre-transplant therapy. The only study that has addressed these investigated patients with mostly incident tumors and reported a five-year recurrence-free survival of up to 50% in patients with intrahepatic and hilar cholangiocarcinoma [15]. This data has suggested that liver transplantation is an option for intrahepatic cholangiocarcinoma management. Nevertheless, the criteria for patient selection have not been elucidated in the literature.

The prevalence of cholangiocarcinoma has increased on a global scale in the past three decades, especially intrahepatic cholangiocarcinoma. However, the most common biliary tract cancer in developed countries is hilar cholangiocarcinoma. The overall survival of intrahepatic cholangiocarcinoma is less than one year after receiving systemic therapy due to late detection of tumors at an advanced stage where it is unresectable [28]. Consequently, other treatment modalities need to be considered for the management of these patients. Hilar cholangiocarcinoma is highly responsive to liver transplantation and has been approved the benefit of neoadjuvant therapy. Nevertheless, previous studies showed that intrahepatic cholangiocarcinoma followed by liver transplantation has a poor response. A recognized risk factor for recurrence of intrahepatic cholangiocarcinoma followed by liver resection is tumor size. Multiple studies have shown that tumor size is correlated with tumor recurrence after liver transplantation, One retrospective, multicenter cohort study in Spain reported the overall survival for intrahepatic cholangiocarcinoma with a cumulative radiographic diameter of less than 8 cm was up to 78% at one year, 66% at three years and 51% at five years [29]. Correspondingly, a multi-national retrospective study showed that early detected (<2 cm) tumors five-year overall survival rate was 80% in contrast with intermediate tumors (2–3 cm) with 61% and 42% for advanced tumors (>3 cm) [10]. A case series study in China analyzed patients with intrahepatic cholangiocarcinoma with liver transplantation managed by liver resection or pre-transplant loco-regional therapy and reported that tumor recurrence was linked with tumor size greater than 5 cm [30].

According to selection criteria, degradation of the disease or its stability was mandatory for at least six months before transplantation in our case series. The overall survival of intrahepatic cholangiocarcinoma was significantly enhanced with transplantation compared to liver resection (83% versus 25% in a preceding study). However, 10% of candidates in our study had recurrent diseases.

Our case series has the capability to pilot for the development of a larger clinical trial aimed at investigating liver transplantation treatment for locally advanced intrahepatic cholangiocarcinoma. Regarding this clinical trial, careful attention should be paid to the presence of lethargic disease, particularly the necessity of continuous neoadjuvant therapy before liver transplantation.

Using the results from a randomized controlled trial, for instance, the ABC-01 study [24] conducted in the UK in patients with advanced or metastatic cholangiocarcinoma or other biliary tract tumors treated with Gemcitabine alone or in combination with cisplatin showed that overall survival was up to 11 months. However, few patients were alive after five years [31].

Most patients with intrahepatic cholangiocarcinoma treated by liver resection who are alive at five years have the early-stage disease and comprise only 20–40% of patients. This analysis was supported by a study of a total of 933 patients who had intrahepatic cholangiocarcinoma subjected to liver resection. Results showed that up to 73% of patients experienced a recurrence of intrahepatic cholangiocarcinoma after liver resection with a five-year overall survival rate of 41%. In multifarious inspection, they found that the risk of intrahepatic recurrence was linked to tumor pathologic features such as a number of lesions, tumor size (>5 cm in diameter), and the presence of satellite lesions.

Here, all transplanted patients had a high risk of recurrence after tumor resection. The five-year overall survival for patients with intrahepatic cholangiocarcinoma has infrequently been more than 80% regardless of tumor resection or chemotherapy. Our conclusions that no full response occurred to transplanted patients in addition to the risk of recurrence after resection in one patient attests to the aggressive nature of the disease. Regarding to the effect of liver fibrosis on survival of patients with IHCCA who receiving gemcitabine chemotherapy, Kinzler et al. [32] were addressing that fibrosis has no significant impact on OS and PFS as well as that patients should not be prevented from state-of-the-art chemotherapy.

Liver toxicity due to chemotherapy is likely to occur in untransplanted patients and should be an important consideration. However, gemcitabine can be dose adjusted to 800 mg/m^2^ if total bilirubin is greater than 1.6 mg/dL and if tolerated, maybe escalated back to 1000 mg/m^2^. Cisplatin is not metabolized by the liver. Of note, none of the subjects in this study could have had stable disease on medical therapy or would have been being downstaged for surgical resection after medical therapy.

However, the clinical trial which employed gemcitabine and cisplatin, and nab-paclitaxel for the management of advanced intrahepatic cholangiocarcinoma indicated that around 10–20% of patients with the unresectable disease were downstaged for tumor resection and 50% had the capability for responding to treatment or having stable disease.

Our case series result suggests that liver transplantation is preferred over liver resection and other modalities such as radical hepatobiliary techniques for advanced intrahepatic cholangiocarcinoma resection, hepatic artery and portal vein remodeling, hepatic vein reimplantation, and in situ cold perfusion. Consequently, we opine that patients who respond to treatment or have the stable disease may benefit from liver transplantation.

By reviewing genomic profiles for biliary tract cancers and their implications for clinical practice, we conclude that the patient outcomes are largely influenced by tumor biology and should guide decision-making regarding who may benefit from sustained response to chemotherapy or targeted therapy. Improvements in bioinformatics and genomic profiling have largely expanded our knowledge of genetic mutations which frequently occur in intrahepatic cholangiocarcinoma [33,34,35].

We acknowledge the limitations of our findings from this case series from an external validity perspective. Nevertheless, our well-designed study is hypothesis-generating and contributes data to real-world management of intrahepatic cholangiocarcinoma as it relates to liver transplantation. Our study results indicate and highlight the requirement for a large size number, multicenter, prospective trial of neoadjuvant therapy followed by liver transplantation for intrahepatic cholangiocarcinoma in the future.

## 5. Conclusions

Our study illustrated that gemcitabine and cisplatin as neo-adjuvant therapy with no radiation resulted in excellent outcomes for patients with intrahepatic and hilar cholangiocarcinoma who underwent liver transplantation. A prospective clinical trial of gemcitabine and cisplatin as neoadjuvant therapy followed by liver transplantation for intrahepatic cholangiocarcinoma is highly needed to confirm these outcomes.

## Figures and Tables

**Table 1:** Patients’ details who received gemcitabine plus cisplatin as a neo-adjuvant treatment for cholangiocarcinoma prior to liver transplantation with their related outcomes.

Patients ID	Sex	Native Liver Diagnosis	Treatment Duration- Days	Days to Transplant	Recurrence or Rejection	Days to The Date of Recurrence or Rejection	Days to The Last Follow up	Days to Death
1	Female	HCCA	603	8	No		813	
2	Male	IHCCA	149	5	Yes	603	871	885
3	Male	IHCCA	250	20	No		824	
4	Male	IHCCA	120	369	No		967	
5	Male	IHCCA	83	472	No		1405	
6	Male	HCCA	161	64	No		418	
7	Male	HCCA	201	5	No		812	
8	Male	IHCCA	206	79	No		831	
9	Male	IHCCA	77	445	No		1834	
10	Female	IHCCA	200	113	No		870	

**Table 2:** Transplant-related outcomes in patients who received Gemcitabine plus Cisplatin as a neo-adjuvant treatment for cholangiocarcinoma prior to liver transplantation; data were presented as median (25th percentile–75th percentile) for continuous variables and number (%) for categorical variables.

The Basic Characteristics of the Included Patients Who Received Gemcitabine Plus Cisplatin as a Neo-Adjuvant Treatment for Cholangiocarcinoma Prior to Liver Transplantation	
Total	
*N* = 10	
Age	
62.71 (60.02–71.87)	
Gender	
Female	2 (20.00)
Male	8 (80.00)
Race	
Asian	1 (10.00)
Black	1 (10.00)
Caucasian	8 (80.00)
Ethnicity	
Hispanic or Latino	2 (20.00)
Not Hispanic or Latino	8 (80.00)
Recurrence or rejection	
Yes	1 (10.00)
no	9 (90.00)
Recurrent time	612.00 (612.00–612.00)
Days for GIM/CIS	181.00 (120.00–250.00)
Death	
0	9 (90.00)
1	1 (10.00)
Follow-up time (days)	
851.00 (813.00–967.00)	

## Data Availability

The data presented in this study are available on request from the corresponding author. The data are not publicly available due to patient confidentiality.
